# Telomere Length and Genetic Variations in Acquired Pediatric Aplastic Anemia: A Flow-FISH Study in Korean Patients

**DOI:** 10.3390/diagnostics15070931

**Published:** 2025-04-04

**Authors:** Yuna Hong, Jong-Mi Lee, Chaeyeon Lee, Duyeon Na, Jin Jung, Ari Ahn, Jae Won Yoo, Jae Wook Lee, Nack-Gyun Chung, Myungshin Kim, Yonggoo Kim

**Affiliations:** 1 Catholic Genetic Laboratory Center, Seoul St. Mary’s Hospital, College of Medicine, The Catholic University of Korea, Seoul 06591, Republic of Korea; yuna1013@catholic.ac.kr (Y.H.); jongmi1226@catholic.ac.kr (J.-M.L.); lcywin0813@catholic.ac.kr (C.L.); msna09@catholic.ac.kr (D.N.); yonggoo@catholic.ac.kr (Y.K.); 2 Department of Medical Sciences, Graduate School of The Catholic University of Korea, Seoul 06591, Republic of Korea; 3 Department of Laboratory Medicine, College of Medicine, The Catholic University of Korea, Seoul 06591, Republic of Korea; bluejin1227@gmail.com; 4 Department of Laboratory Medicine, Incheon St. Mary’s Hospital, College of Medicine, The Catholic University of Korea, Seoul 06591, Republic of Korea; 0124ari@gmail.com; 5 Department of Pediatrics, Catholic Hematology Hospital, Seoul St. Mary’s Hospital, College of Medicine, The Catholic University of Korea, Seoul 06591, Republic of Korea; hoiring0209@gmail.com (J.W.Y.); dashwood@catholic.ac.kr (J.W.L.)

**Keywords:** aplastic anemia, telomere length, flow-fluorescence in situ hybridization

## Abstract

**Background**: Aplastic anemia (AA) is a rare bone marrow failure syndrome characterized by notably short telomere length, which is associated with treatment responses. In this study, we measured telomere lengths in Korean pediatric AA patients using flow-fluorescence in situ hybridization (Flow-FISH) and explored their shortening in relation to disease characteristics, genetic conditions and patient outcomes. **Methods**: We analyzed peripheral blood samples from 75 AA patients and 101 healthy controls. Telomere lengths were measured using Flow-FISH, and relative telomere length (RTL) and delta RTL assessments were conducted. Genetic evaluations included karyotyping, chromosome breakage tests and clinical exome sequencing (CES) to identify inherited bone marrow failure syndrome (IBMFS)-associated genetic variants. **Results**: Telomere lengths in AA patients were significantly lower than those of age-adjusted healthy controls. Patients receiving immunosuppressive therapy tended to have long telomeres, as indicated by high delta RTL values. Patients with genetic abnormalities, including karyotype abnormalities (*n* = 2) and genetic variants (*n* = 11) such as carrier genes of IBMFS or variants of unclear significance, showed significantly short telomere lengths. **Conclusions**: This study reinforces the importance of telomere length as a biomarker in acquired AA. Utilizing Flow-FISH, we were able to accurately measure telomere lengths and establish confidence in this method as an appropriate laboratory test. We found significant reduction in telomere lengths in AA patients, and importantly, longer telomeres were correlated with better outcomes in immunosuppressive therapy. Additionally, our genetic analysis underscored the relevance of variants in IBMFS-associated genes to the pathophysiology of short telomeres.

## 1. Introduction

Aplastic anemia (AA) is a rare and life-threatening bone marrow failure syndrome (BMFS) characterized by peripheral blood pancytopenia and a significant reduction in bone marrow hematopoietic cell proliferation [1,2]. In complete blood count (CBC), AA presents with pancytopenia, reticulocytopenia, and normochromic, normocytic, or macrocytic anemia. Among these patients, severe aplastic anemia (SAA) is diagnosed when either bone marrow cellularity is less than 25% or between 25% and 50% with fewer than 30% residual hematopoietic cells, and at least two of the following criteria are met: a neutrophil count of less than 500 × 10^9^/L, a platelet count of less than 20 × 10^9^/L, or an absolute reticulocyte count of less than 60 × 10^9^/L. Furthermore, an absolute neutrophil count of less than 0.2 × 10^9^/L qualifies for a diagnosis of very severe aplastic anemia (VSAA). Patients who do not meet the criteria for both SAA and VSAA are classified as non-severe aplastic anemia (NSAA) [3]. AA has an incidence of 5.16 cases per 1 million people per year in South Korea, approximately 2–3 times higher than the 2 cases per 1 million people per year reported in Western countries [4]. While both hereditary and acquired forms of AA are recognized, the acquired form is more prevalent. The hereditary form of AA is typically associated with genetic mutations inherited from one or both parents, leading to inherited bone marrow failure syndromes (IBMFS), which are often linked with other genetic or developmental abnormalities, such as Fanconi anemia (FA), Shwachman–Diamond syndrome, and Dyskeratosis congenita (DC). In contrast, acquired AA generally does not present with additional genetic or developmental abnormalities [5]. Acquired AA typically results from immune-mediated destruction of hematopoietic stem cells, with environmental factors such as exposure to certain chemicals, drugs and viral infections often serving as triggers. The diagnosis of AA relies on bone marrow (BM) examination.

Differentiating between IBMFS and acquired forms is essential for optimal treatment strategies [6,7]. Although a family history or characteristic physical abnormalities may suggest IBMFS, cryptic or atypical presentations can complicate the diagnosis [8]. Several consensus guidelines for the diagnosis of BMFS in children describe the usefulness of a comprehensive diagnostic work-up [9,10]. This approach combines a detailed interview, encompassing both family and present medical histories, with a physical examination, blood tests and BM evaluations. It also includes genetic analyses and screening tests designed to exclude IBMFS, such as chromosome fragility test for FA and telomere length measurement by flow fluorescence in situ hybridization (Flow-FISH) for DC [11,12].

Telomeres, protective caps at the ends of chromosomes, are made up of repetitive nucleotide sequences that safeguard genomic DNA from erosion during cell division. These structures naturally shorten with each cell division, serving as a biological clock that ultimately limits the lifespan of a cell. Research indicates that patients with AA often have significantly shorter telomeres in hematopoietic cells than those of healthy individuals [13]. While individuals with DC exhibit significantly short telomeres, it is noted that 30 to 50% of those with acquired AA also present with shortened telomeres [14,15], a condition linked with increased risks of relapse, clonal evolution and decreased survival rates after immunosuppressive treatment. Therefore, a study on telomere shortening in patients with AA not only provides insight into the disease’s pathogenesis but also hints at potential areas for therapeutic exploration, suggesting that targeting telomere dynamics could offer new strategies for treatment.

In this study, we measured telomere length in peripheral blood mononuclear cells from Korean pediatric patients with acquired AA using Flow-FISH analysis. Our aim was to assess telomere shortening within this group and explore whether telomere length is associated with clinical factors and any genetic variants incidentally identified in genetic studies. Furthermore, we sought to determine the suitability of Flow-FISH for clinical laboratory tests in indicated individuals.

## 2. Materials and Methods

### 2.1. Sample

We analyzed peripheral blood samples collected from 75 patients diagnosed with acquired AA at Seoul St. Mary’s Hospital. The diagnosis and severity of AA were confirmed based on CBC and bone marrow biopsy criteria, as described by the North American Pediatric Aplastic Anemia Consortium [3]. Patients who tested positive for an IBMFS mutation or had a positive chromosome breakage test were not included. Of the 75 patients, 50 were monitored without treatment and had samples available either at diagnosis or from an untreated state. Among those who underwent treatment, samples from 11 patients were collected after immunosuppressive therapy (IST). A literature review of previous studies found no evidence that IST influences telomere length; therefore, these patients were included in the study. The remaining 14 patients received HSCT, and all had samples taken before transplantation. We additionally obtained control peripheral blood samples from 101 individuals, ranging in ages from 0 to 100 years, who visited Seoul St. Mary’s Hospital for routine health check-ups or outpatient care. Only those without hematologic abnormalities or diseases associated with short telomeres were included to establish a normal regression line. Since telomere length decreases with age, all telomere length assessments were conducted based on the patients’ age at the time of sample collection. Informed consent for blood sampling was obtained according to a protocol approved by the Institutional Review Board of the Seoul St. Mary’s Hospital (No. KC23SISI0219).

### 2.2. Telomere Length Measurement Using Flow-FISH

Telomere length was measured using Flow-FISH with PNA Kit/FITC (Agilent Technologies, Singapore, Singapore), according to the instruction of the manufacturer. Peripheral blood mononuclear cells (PBMCs) were isolated from fresh whole blood through Ficoll (Merck, Darmstadt, Germany) density centrifugation. The 1301 cell line (human T-cell leukemia) purchased from ECACC (European Collection of Authenticated Cell Cultures, Salisbury, UK) were used as a control. Sample and control cells were prepared by mixing 2 × 10^6^ of each, diluting the mixture to 6 mL with DPBS (Dulbecco’s Phosphate Buffered Saline) and dividing it into four labeled microcentrifuge tubes (Sarstedt, Nümbrecht, Germany) (A, B, C and D), each containing 1.5 mL. The cells were then centrifuged to remove the supernatant. DNA denaturation occurred at 82 °C for 10 min in a microcentrifuge tube containing hybridization solution, with fluorescein-conjugated Peptide Nucleic Acid (PNA) telomere probe added to tubes A and B but not to tubes C and D. Hybridization was conducted in the dark at room temperature overnight, followed by two washes with wash solution at 40 °C for 10 min each. Cells were subsequently resuspended in DNA-staining solution and stored in the dark at 2 to 8 °C for two to three hours before flow cytometry analysis. Fluorescence analysis was performed using a FACSLyric flow cytometer (BD Biosciences, Franklin Lakes, NJ, USA). At least 20,000 cells were acquired and analyzed. Fluorescence from telomere staining was observed in channel FL1, and from DNA staining in channel FL3.

Telomere length analysis was performed without knowledge of demographic or clinical characteristics, and sequential samples from the same patient were excluded from the analysis. Relative telomere length (RTL) was calculated using the formula as follows:

$$R\mathrm{TL}=\frac{(\mathrm{mean}\;\mathrm{FL}1\;\mathrm{of}\;\mathrm{sample}\;\mathrm{cells}\;\mathrm{with}\;\mathrm{probe}\;-\;\mathrm{mean}\;\mathrm{FL}1\;\mathrm{of}\;\mathrm{sample}\;\mathrm{cells}\;\mathrm{without}\;\mathrm{probe})\;\times \;\mathrm{DNA}\;\mathrm{index}\;\mathrm{of}\;\mathrm{control}\;\mathrm{cells}\;\times \;100}{(\mathrm{mean}\;\mathrm{FL}1\;\mathrm{of}\;\mathrm{control}\;\mathrm{cells}\;\mathrm{with}\;\mathrm{probe}\;-\;\mathrm{mean}\;\mathrm{FL}1\;\mathrm{of}\;\mathrm{control}\;\mathrm{cells}\;\mathrm{without}\;\mathrm{probe})\;\times \;\mathrm{DNA}\;\mathrm{index}\;\mathrm{of}\;\mathrm{sample}\;\mathrm{cells}}$$

We used delta RTL to compare telomere lengths of patients with those of age-matched healthy controls [16]. Delta RTL was calculated as the difference between RTL and age-adjusted RTL derived from the healthy controls.

### 2.3. Genetic Evaluation

Karyotyping was conducted in 65 patients, and a chromosome breakage test was performed to screen for FA in 48 of these patients [17,18]. In addition, clinical exome sequencing (CES) was carried out to identify the IBMFS-associated genetic variants in 42 patients using the TruSightOne Expanded kit (Illumina, San Diego, CA, USA) [19]. Genomic DNA extracted from peripheral blood was used to construct a library. The concentrated library fragments were then amplified and sequenced using a Nextseq 550 system (Illumina, San Diego, CA, USA). All detected variants were classified according to the standards and guidelines set by the American College of Medical Genetics and Genomics and the Association for Molecular Pathology [20].

### 2.4. Statistical Analysis

Our data were presented as the mean ± standard deviation (SD) or as median values with ranges indicated in the tables. Pearson’s correlation coefficient was utilized to assess the association between RTL and age. The Mann–Whitney U test was employed to compare RTL and delta RTL between subgroups. All *p*-values were two-sided, with values of 0.05 or less denoting statistical significance. Statistical analyses were conducted using IBM SPSS software, version 24 and R statistical software, version 4.3.3.

## 3. Results

### 3.1. Patients

The median age of patients at the time of diagnosis was 9 years, with ages ranging from 1 to 17 years (Table 1). Among our subjects, 51 patients were diagnosed with NSAA (68%), 19 with SAA and five with VSAA (32%). While 50 patients were followed up regularly without treatment, 11 patients were treated with IST with anti-thymocyte globulin or anti-lymphocyte globulin ± cyclosporin A, and 14 patients underwent HSCT. Among 11 patients treated with IST, four showed a complete response, two showed a partial response, and five were non-responders [21] (Table 1). As a control, samples were collected from healthy individuals with normal blood cell counts, ranging from 0 to 100 years of age. Twenty of these individuals were under the age of 19.

### 3.2. Telomere Length of Patients with AA

**Figure 1:**
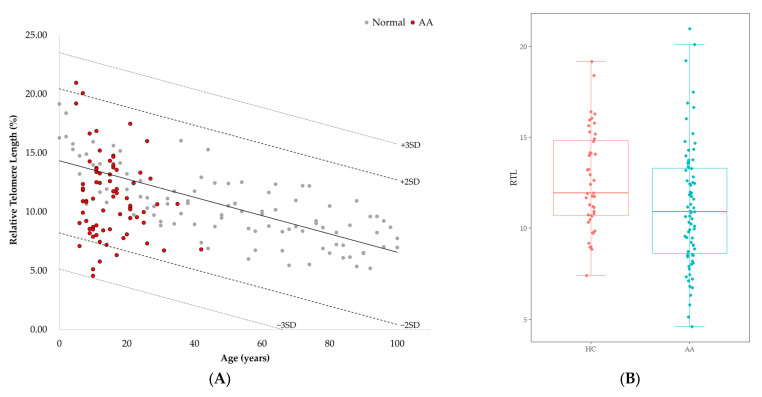
(**A**) Telomere length according to age measured by Flow-FISH. Gray dots represent healthy controls (*n* = 101). Red dots represent AA patients (*n* = 75). The healthy control is denoted as mean (2SD, 3SD) as a black line (dotted lines). Linear regression plot: y = −0.0776x + 14.332 (*p* < 0.001). (**B**) Comparison of RTL between age-adjusted health controls (HC) and AA patients (*p* = 0.010).

**Figure 2:**
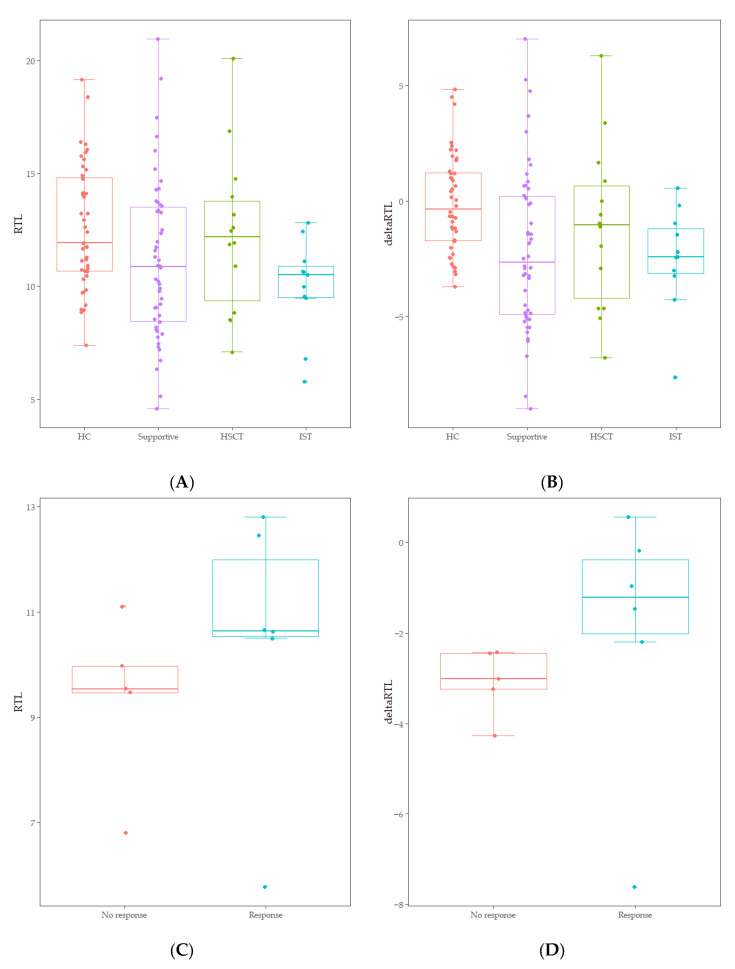
(**A**) Comparison of RTL between age-adjusted healthy controls (HC) and treatment groups (Supportive, HSCT, IST) (*p* = 0.060). (**B**) Comparison of delta RTL between age-adjusted healthy controls (HC) and treatment groups (Supportive, HSCT, IST) (*p* = 0.013). (**C**) Comparison of RTL between non-responders and responders within IST treatment groups (*p* = 0.201). (**D**) Comparison of delta RTL between non-responders and responders within IST treatment groups (*p* = 0.068).

RTL of healthy controls showed a significant negative correlation with age (r = −0.744, *p* < 0.001) (Figure 1). The median value of RTL was 11.92 (range: 7.38–19.16) in age-adjusted healthy controls, which was significantly higher than that in AA patients [10.89 (range: 4.57–20.96); *p* = 0.010] (Figure 1). Five out of 75 AA patients (6.7%) displayed RTL values that were more than two SDs below those of age-adjusted healthy controls, and none were more than three SDs below (Figure 1). Delta RTL was expressed as the distance from the age-appropriate point on the normal regression line of RTL versus age. The SD for delta RTL in healthy control was 2.04. The median value of delta RTL was −2.39% (range: −8.99–7.02%) in AA patients. There were no significant differences in RTL or delta RTL across varying severities of disease or types of treatment. Among patients who received IST, the median delta RTL in responders tended to be higher than that in non-responders [−1.21 (range: −7.62–0.57) vs. −3.01 (range: −4.27–−2.42), *p* = 0.068] (Figure 2).

### 3.3. Telomere Length and Genetic Variants

**Figure 3:**
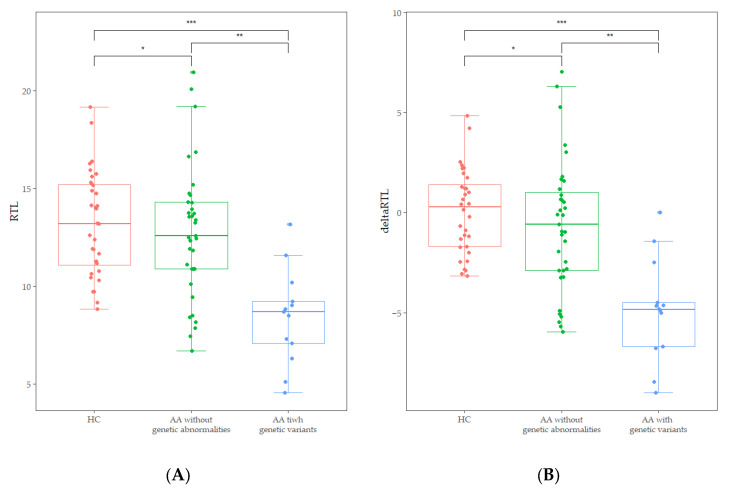
(**A**) Comparison of RTL between age-adjusted healthy controls (HC), AA patients without genetic abnormalities, and those with IBMFS-associated variants and abnormal karyotypes. (* *p* = 0.448, ** *p* < 0.001, *** *p* < 0.001). (**B**) Comparison of delta RTL between age-adjusted healthy controls (HC), AA patients without genetic abnormalities, and those with IBMFS-associated variants and abnormal karyotypes (* *p* = 0.228, ** *p* = 0.001, *** *p* < 0.002).

Abnormal karyotypes were detected in two patients; a deletion of the long arm of chromosome 20 and a duplication of the long arm of chromosome 1. The chromosome breakage test revealed that no patients exhibited values above the cut-off of 1.0. Out of the results of 48 patients who underwent CES, four pathogenic variants and one likely pathogenic variant were identified. Additionally, seven variants of uncertain significance were identified (Table 2). Two variants were present in one patient, and each of the other variants was present in different patients. However, none was genetically diagnosed with IBMFS, because the presence of a pathogenic or likely pathogenic variant was identified as a single heterozygous carrier status in an autosomal recessive disorder, not in a compound heterozygous status. FA-associated genetic variants were detected in three patients, DC in two patients, and AA in two patients. Interestingly, patients with IBMFS-associated variants or abnormal karyotypes (*n* = 13) showed significantly lower RTL [8.70 (4.57–13.17 vs. 12.59 (6.72–20.96), *p* < 0.001)] and delta RTL [−4.83 (−8.99–0.00) vs. −0.58 (−5.95–7.02), *p* < 0.001] than those of patients without genetic abnormalities (Figure 3). Additionally, the decrease of RTL and delta RTL was not influenced by gender or age.

## 4. Discussion

Telomeres consist of non-coding, repetitive DNA sequences with a 5′-TTAGGG-3′ motif at the ends of linear chromosomes [22]. Abnormalities in telomere biology have been identified in several conditions, such as the initially recognized DC, Hoyeraal–Hreidarsson syndrome, SAA and pulmonary fibrosis [23].

Techniques such as the terminal restriction fragment analysis via Southern blot analysis, quantitative PCR, telomere/centromere FISH and Flow-FISH have been used to measure telomere length [24]. Southern blot analysis, often considered the gold standard for telomere length measurement, directly measures telomere length using restriction enzymes and has been widely used due to its relative accuracy and high reproducibility. However, this method involves a complex experimental process, requires a large amount of DNA, has a long processing time, and is limited in detecting short telomeres [25]. In contrast, qPCR measures the ratio of telomere repeats to single-copy genes (T/S ratio), making it faster, simpler, and requires less DNA than Southern blot [26]. Flow-FISH, based on FISH technology, uses PNA to measure fluorescent signal intensity and allows for telomere length measurement by individual cell subtypes without the need for DNA extraction. While the procedure is simpler than Southern blot analysis, it maintains a high degree of correlation [27,28]. Flow-FISH also offers superior accuracy, precision, and reproducibility. In particular, a direct comparison of qPCR and Flow-FISH showed that Flow-FISH had higher sensitivity and specificity due to its ability to measure telomere length at the single-cell level, reducing the risk of false positives and false negatives seen in qPCR. Flow-FISH also provides more consistent results in repeat measurements, making it a more reliable method for telomere length analysis [29].

In this study, we successfully established Flow-FISH in patients with AA and deduced two variables to express telomere length in comparison with age-matched healthy controls. We adopted the Telomere PNA kit to detect telomere sequences in cell suspensions via flow cytometry. The procedure involves denaturing sample DNA with or without a fluorescein-conjugated PNA telomere probe, followed by overnight hybridization at room temperature. After two washes, the cells were prepared for flow cytometry analysis, optionally using DNA Staining Solution for cell cycle analysis. RTL was calculated by comparing the telomere signal in sample cells to that in control cells, adjusted for DNA content using propidium iodide staining. Both RTL and delta RTL values were used to represent telomere length, with delta RTL providing an advantage due to age-matching [16]. As laboratory tests, each laboratory obtained data from healthy controls to establish the normal regression line of RTL versus age to calculate delta RTL.

According to our data, telomere length declined with age and was significantly short in AA patients, though not influenced by disease severity. Our study is consistent with previous studies that reported shortened telomeres in 30 to 50% of patients with acquired AA [15,30]. Longer telomeres also exhibited a better response to IST, serving as a significant predictor of IST outcomes [16,31]. Telomere length can provide important information for patients undergoing IST or preparing for HSCT. Through such analysis, it is also vital to confirm that related donors are healthy and unaffected by potentially heritable disease [32]. Studies have indicated that long donor telomeres, independent of donor age, are associated with improved survival after HSCT [33,34,35]. Both RTL and delta RTL represent telomere length; however, delta RTL provides more a straightforward comparison of telomere lengths than that of age-matched healthy control and differences in AA patients.

Patients with genetic abnormalities, including karyotype abnormalities and IBMFS-associated variants, exhibited short telomere lengths. The presence of genetic variants indicates either a carrier state of IBMFS or unclear significance of the variant. Among these genetic variants, two were associated with telomere biology disorder (*TERT* and *WRAP55*), and two were associated with FA (*BRCA2*). Although most studies do not associate the presence of heterozygous FA-associated variants with the development of BMF or increased cancer risk, evidence suggests that heterozygous FA gene carriers may face delayed onset of and increased risk for myelodysplastic neoplasm and acute myeloid leukemia, indicating that normal alleles may not provide complete protection [36]. The FA-associated significant variations were enriched in AA and hematologic malignancies, which suggests that heterozygous mutations of FA genes contribute to hematopoietic failure and leukemogenesis [37]. Current studies have yet to understand why AA patients with genetic variants exhibit short telomeres. We hypothesize that such short telomeres could result from increased telomere attrition due to the rapid turnover of hematopoietic cells in response to bone marrow stress [38]. We also cannot dismiss the possibility of unidentified genotypes associated with the risk of telomere shortening or acquired AA [39,40,41].

There are some limitations to this study. First, the sample size used in the research may not have been large enough, which could impact the generalizability of the results. Second, patients with AA can have a variety of causes and clinical characteristics, which may introduce heterogeneity among them [42]. Third, the limited sample availability prevented the CES analysis from being performed on all patients. Additionally, while we explored the relationship between some IBMFS-related genetic mutations and telomere shortening, the correlation remains incompletely understood.

This study reinforces the importance of telomere length as a biomarker in acquired AA. Utilizing Flow-FISH, we were able to accurately measure telomere lengths and establish confidence in this method as an appropriate laboratory test. We found significant reduction in telomere lengths in AA patients, and importantly, longer telomeres were correlated with better outcomes of IST. Additionally, our genetic analysis underscored the relevance of variants in IBMFS-associated genes in the pathophysiology of short telomeres. Future research should focus on integrating telomere length and genetic abnormalities into clinical practice using reliable measurement techniques.

## Figures and Tables

**Table 1:** Clinical characteristics and telomere changes of aplastic anemia patients.

Patient Subgroups	Number (%)	Age, Years *	Age at Diagnosis, Years	Sex, Male	ANC (×10^9^/L)	Hb (g/dL)	Platelet (×10^9^/L)	RTL	Delta RTL
Total	75 (100)	13 (5–42)	9 (1–17)	43	0.92 (0.05–1.94)	9.7 (4.7–12.7)	36.0 (5–84)	10.89 (4.57–19.20)	−0.34 (−3.69–4.83)
Severity									
NSAA	51 (68.0)	14 (5–31)	9 (2–16)	29	1.10 (0.08–1.94)	10.6 (4.7–12.7)	41 (10–84)	10.63 (4.57–19.20)	−2.42 (−8.99–5.26)
SAA	19 (25.3)	11 (5–42)	7 (4–17)	11	0.68 (0.27–1.94)	8.2 (5.3–10.4)	16 (5–50)	11.11 (6.80–20.96)	−1.95 (−4.65–7.02)
VSAA	5 (6.7)	12 (6–17)	10 (1–17)	3	0.15 (0.05–0.26)	8.2 (6.2–9.5)	11 (7–16)	8.50 (5.78–13.96)	−5.06 (−7.32–0.87)
Treatment									
Supportive	50 (66.7)	12 (5–31)	9 (3–16)	29	1.03 (0.08–1.94)	10.5 (4.7–12.7)	40 (10–84)	10.86 (4.57–20.96)	−2.65 (−8.99–7.02)
IST									
Responder	6 (8.0)	25 (12–35)	7 (1–15)	2	0.90 (0.15–1.18)	9.1 (7.7–12.3)	36 (11–48)	10.65 (5.78–12.81)	−1.21 (−7.62–0.57)
Non-responder	5 (6.7)	23 (10–42)	12 (5–17)	2	1.10 (0.31–1.94)	8.6 (5.3–11.4)	32 (7–80)	9.54 (6.80–11.11)	−3.01 (−4.27–−2.42)
HSCT	14 (18.7)	12 (6–17)	9 (4–17)	10	0.44 (0.05–1.50)	8.2 (6.2–12.1)	15 (5–50)	12.19 (7.09–20.08)	−1.02 (−6.78–6.29)
Genetics									
Variants (+)	13 (27.1)	11 (6–26)	8 (3–16)	10	1.30 (0.26–1.62)	9.9 (4.7–12.4)	44 (7–62)	8.70 (4.57–13.17)	−4.83 (−8.99–0.00)
Variants (–)	35 (72.9)	11 (5–31)	9 (4–17)	21	0.83 (0.05–1.87)	9.3 (6.2–12.7)	32 (5–84)	12.59 (6.72–20.96)	−0.58 (−5.95–7.02)

* Age at the time of sample collection. Abbreviations: ANC, absolute neutrophil count; RTL, relative telomere length; NSAA, non-severe aplastic anemia; SAA, severe aplastic anemia; VSAA, very severe aplastic anemia; IST, immunosuppressive therapy; HSCT, Hematopoietic Stem Cell Transplantation

**Table 2:** Identified genetic variants.

Gene	Nucleotide Change	Amino Acid Change	OMIM	Transcript	Functional Impact	ID
*BRCA2*	c.1399A>T	p.(Lys467*)	Fanconi anemia, complementation group D1, (AR); Breast-ovarian cancer, familial, 2, (AD)	NM_000059.3	Pathogenic	8
*CDAN1*	c.3124C>T	p.(Arg1042Trp)	Dyserythropoietic anemia, congenital, type Ia (AR)	NM_138477.2	Pathogenic	57
*SLC37A5*	c.1042_1043del	p.(Leu348ValfsTer53)	Congenital disorder of glycosylation, type IIw, (AD)	NM_001164277.1	Pathogenic	73
*MPL*	c.1025del	p.(Pro342GlnfsTer27)	Thrombocythemia 2	NM_005373.2	Pathogenic	74
*PRF1*	c.65delC	p.(Pro22ArgfsTer29)	Aplastic anemia; Hemophagocytic lymphohistiocytosis, familial, 2, (AR); Lymphoma, non-Hodgkin	NM_001083116.1	Likely Pathogenic	18
*WRAP55*	c.914C>T	p.(Thr305Met)	Dyskeratosis congenita, autosomal recessive 3 (AR)	NM_018081.2	VUS	2
*JAGN1*	c.504G>T	p.(Lys168ASN)	Neutropenia, severe congenital, 6, autosomal recessive, 616022, (AR)	NM_032492.3	VUS	17
*BRIP1*	c.2258A>G	p.(Asp753Gly)	Fanconi anemia, complementation group J; {Breast cancer, early-onset, susceptibility to}, (AD, SMu)	NM_032043.2	VUS	18
*NBN*	c.191C>G	p.(Pro64Arg)	Aplastic anemia; Leukemia, acute lymphoblastic; Nijmegen breakage syndrome, (AR)	NM_002485.4	VUS	24
*TERT*	c.2795G>A	p.(gly932ASP)	Dyskeratosis congenita, autosomal dominant 2, (AD); Dyskeratosis congenita, autosomal recessive 4, (AR); Pulmonary fibrosis and/or bone marrow failure syndrome, telomere-related, 1, (AD)	NM_198253.3	VUS	48
*BRCA2*	c.4568G>T	p.(Gly1523Val)	Fanconi anemia, complementation group D1, (AR)	NM_000059.3	VUS	50
*WAS*	c.1130G>A	p.(Arg377His)	Neutropenia, severe congenital; Thrombocytopenia; Wiskott–Aldrich syndrome	NM_000377.3	VUS	51

Abbreviations: OMIM, online Mendelian inheritance in man; AR, autosomal recessive; AD, autosomal dominant; SMu, somatic mutation; VUS, variant uncertain significance.

## Data Availability

The raw data supporting the conclusions of this article will be made available by the authors on request.
